# Knowledge, attitudes, and prevalence of vitamin C intake among the general population of the UAE during the COVID-19 pandemic

**DOI:** 10.3389/fpubh.2025.1648213

**Published:** 2025-09-05

**Authors:** Balsam Qubais Saeed, Ali Al Janaahi, Ashmita Pinheiro, Aisha Al Shamsi, Shaikha Salah Alhaj, Ahmad Omar Adrees, Hind Al Maeeni, Razan Darwish, Ahmed Hammad, Noor Sarchanar Jamal, Mahmood Haitham Al Awaad

**Affiliations:** ^1^Department of Clinical Sciences, College of Medicine, University of Sharjah, Sharjah, United Arab Emirates; ^2^Sharjah Institute for Medical Research, University of Sharjah, Sharjah, United Arab Emirates; ^3^College of Medicine, University of Sharjah, Sharjah, United Arab Emirates; ^4^Mohammad Bin Rashid University, Dubai Health, Dubai, United Arab Emirates

**Keywords:** vitamin C, COVID-19, knowledge, attitudes, prevalence, United Arab Emirates (UAE), public health, cross-sectional study

## Abstract

**Background:**

The extent of vitamin C (VC) deficiency and the general intake of this vitamin among the population is currently unknown. Therefore, in this study, we aimed to evaluate the knowledge, attitudes, and prevalence of vitamin C intake among the general population in the United Arab Emirates (UAE).

**Methods:**

A cross-sectional study was conducted between February and March 2022 among adults aged 18 years and above residing in the UAE. Participants were recruited through an online platform using a voluntary response sampling approach. Data were collected using a pre-designed, self-administered, 22-item questionnaire available. It assessed four domains: demographics, knowledge, practices, and attitudes related to vitamin C consumption. Data were analyzed using SPSS version 26. Descriptive statistics were computed, and chi-square tests were used to evaluate associations between demographic factors and knowledge levels. A *p*-value of < 0.05 was considered statistically significant.

**Results:**

Among 720 participants, 86.1% were female, and 75.6% were aged 18–29. Despite 88.2% reporting VC consumption, only 30.1% demonstrated adequate knowledge, with significant age-related differences (*p* = 0.015). Younger participants had significantly higher knowledge scores than other age groups. During the COVID-19 pandemic, 53.5% of previous non-consumers began using VC. Attitudes varied 85.8% believed VC aids COVID-19 recovery, 45.7% thought it prevents infection, and 81.9% acknowledged the potential harm from overconsumption. Pandemic-related health concerns significantly increased VC intake (*p* < 0.001).

**Conclusion:**

There is a clear need for evidence-based public health strategies to improve awareness and safe practices related to vitamin C consumption in the UAE. Educational interventions delivered through healthcare professionals, especially pharmacists, and supported by media campaigns should aim to correct misconceptions, promote appropriate use, and prevent potential misuse of vitamin C during public health crises.

## 1 Introduction

Vitamin C (VC), or ascorbic acid, is a vital water-soluble nutrient that acts as both a potent antioxidant and an essential cofactor in numerous enzymatic and biosynthetic processes within the human body, particularly those involved in immune defense and collagen formation (1, 2). Since humans cannot synthesize VC endogenously, it must be obtained through dietary intake. VC has long been recognized for its antiviral and anti-inflammatory effects, especially in the context of respiratory illnesses.

Multiple studies have reported that regular VC supplementation may reduce the incidence and duration of the common cold, particularly in individuals under physical stress or with marginal VC status (1, 38). Its potential role in reducing the severity of pneumonia has also been documented. During the COVID-19 pandemic, VC gained renewed attention due to its immunomodulatory properties and potential to mitigate the effects of acute respiratory viral infections, including SARS-CoV-2. VC levels have been observed to decline during acute infections, and intravenous VC has been investigated as a therapeutic agent in hospitalized COVID-19 patients to reduce inflammation, oxidative stress, and cytokine storm severity (3–5).

Vitamin C plays a pivotal role in modulating the immune response, particularly by mitigating cytokine storms and downregulating proinflammatory mediators during the critical stages of COVID-19. Furthermore, it may enhance host resistance to a range of viral infections and help alleviate associated symptoms, including those caused by SARS-CoV-2 (1, 6, 7). Despite these potential benefits, excessive or unregulated use of high-dose VC supplementation presents safety concerns. While VC is generally safe due to its water-soluble nature, high doses have been linked to adverse events such as gastrointestinal disturbances, oxalate nephropathy, and hemolytic anemia, particularly in individuals with glucose-6-phosphate dehydrogenase (G6PD) deficiency (8, 9). Additionally, overreliance on supplements without proper guidance can contribute to a false sense of security, undermining other evidence-based public health measures.

Widespread media coverage of vitamin C during the COVID-19 pandemic, coupled with heightened public interest in preventive health measures, likely influenced patterns of supplement use. However, public perception and health-related decisions are often shaped by misinformation or insufficient knowledge (10, 11). Gaining a clear understanding of the population's knowledge, attitudes, and behaviors regarding vitamin C use particularly during global health emergencies is essential for informing safe, evidence-based public health strategies and interventions (12).

Previous studies conducted in Middle Eastern countries such as Saudi Arabia and Lebanon have reported increased vitamin C consumption during the COVID-19 pandemic (13, 14). However, similar data from the United Arab Emirates (UAE) remain limited. This lack of localized insight restricts the ability of health authorities to design evidence-based awareness campaigns and educational interventions that are tailored to the beliefs, behaviors, and needs of the UAE population. Therefore, this study aims to evaluate the knowledge, attitudes, and prevalence of vitamin C intake among the general adult population in the UAE before and during the COVID-19 pandemic. By identifying trends and gaps, the study seeks to inform public health strategies that promote the rational and informed use of dietary supplements.

## 2 Materials and methods

### 2.1 Study design

This study employed a cross-sectional survey design, conducted in the UAE from all emirates (Abu Dhabi, Dubai, Sharjah, Ajman, Umm Al Quwain, Ras Al Khaimah, and Fujairah), between February 17 and March 19, 2022. A self-administered questionnaire was distributed to adult participants using an online platform. The questionnaire was initially pilot-tested on 15 community members, and necessary revisions were made based on their feedback to enhance clarity and relevance. Two language versions of the questionnaire were developed, Arabic and English, to ensure accessibility for a wider demographic.

The final version consisted of 22 questions, organized into four main domains:

1. Demographics (seven items),
2. Knowledge (six items),
3. Practices (six items), and
4. Attitudes (three items).

Most of the questions were closed-ended, requiring participants to select from predetermined response options.

### 2.2 Study subjects

This cross-sectional study was conducted in Dubai, United Arab Emirates (UAE), targeting the general adult population aged 18 years and above. A total of 1,065 individuals were approached, of whom 720 participants completed the questionnaire and were included in the final analysis.

The inclusion criteria were adults (≥18 years old) residing in UAE who were able to understand and complete the questionnaire in either Arabic or English. Exclusion criteria included individuals under 18 years of age and those unwilling or unable to provide informed consent.

A non-probability sampling method was employed, specifically a volunteer (self-selected) sample recruited through online platforms (e.g., social media, community groups) and public spaces such as shopping malls and community centers. Although the term “random selection” was initially used, we acknowledge that this was not a probability-based random sample but rather a convenience sampling approach.

The minimum required sample size was calculated to be 359 participants, based on a previously reported prevalence of vitamin C use in the region (69.6%), a confidence level of 95%, and a margin of error of 10% (14). Our final sample exceeded this requirement, enhancing the study's statistical power.

### 2.3 Scoring guidelines

The questionnaire consisted of three main sections: knowledge, attitudes, and prevalence related to vitamin C intake.

- Knowledge section: participants responded to 10 multiple-choice questions. Each correct answer was awarded 1 point, and incorrect or “don't know” responses received 0 points. Total scores ranged from 0 to 10. Knowledge levels were categorized as follows: poor (0–3), moderate (4–6), and good (7–10).
- Attitude section: this section included five statements rated on a 5-point Likert scale (strongly disagree to strongly agree). Positive attitudes received higher scores (from 1 to 5), while negative attitudes were reverse scored. The total attitude score ranged from 5 to 25, with higher scores indicating a more favorable attitude toward vitamin C intake.
- Practice section: practices were assessed using descriptive questions regarding frequency and sources of vitamin C intake. These responses were analyzed descriptively and were not converted into numerical scores.

### 2.4 Survey and data collection

The required sample size was calculated based on the smallest demographic subgroup, using a 95% confidence level and a ±5% margin of error, following established survey methodology guidelines (15, 16). A total of 1,065 individuals initially completed the questionnaire via Microsoft Forms, from which 720 eligible responses were retained for final analysis based on inclusion criteria. The survey tool was adapted from previously validated questionnaires (14) to ensure content validity and linguistic clarity, the instrument was reviewed by five academic experts from the University of Sharjah and further pilot-tested by 15 external individuals. Following these validation steps, the questionnaire was administered by a trained team of six researchers in alignment with the study's research objectives and secondary aims.

The online survey included a structured interface that presented the study title, objectives, confidentiality assurance, and instructions. Participants first encountered a consent screen. Upon providing informed consent, they could select their preferred language and proceed to the questionnaire. Both language versions contained identical content and section structure. The questionnaire was divided into five main sections:

Section 1: informed consent;
Section 2: language selection;
Section 3: demographics and vitamin C (VC) intake prior to the COVID-19 pandemic;
Section 4: conditional follow-up on VC use during the pandemic (Sections 2-A for VC users, 2-B for non-users);
Section 5: attitudes toward VC use.

The survey was designed to take approximately 5–10 min to complete. Participation was fully voluntary and anonymous. Participants could withdraw at any point without providing a reason. Those who declined consent were automatically excluded from completing the survey.

### 2.5 Ethics approval

The study received ethical approval from the Research Ethics Committee at the University of Sharjah on February 9, 2022 (Reference No. REC-22-02-09-01-S). All procedures were conducted in accordance with the ethical standards of the Declaration of Helsinki (17).

### 2.6 Data statistics and analysis

Data were analyzed using the Statistical Package for the Social Sciences (SPSS) software, version 26. Descriptive statistics, including frequencies, percentages, means, and standard deviations, were used to summarize participant demographics and responses related to knowledge, attitudes, and practices.

Bivariate analyses were conducted using the chi-square test to examine associations between categorical variables, such as the relationship between demographic factors (e.g., age, gender, education level) and vitamin C intake practices. Independent samples *t*-tests and one-way ANOVA were used to compare mean knowledge and attitude scores across different demographic groups.

A *p*-value of less than 0.05 was considered statistically significant for all analyses. Charts and graphs were created using Microsoft Excel to visually present key findings, but all statistical analyses were performed in SPSS.

## 3 Results

### 3.1 Demographic characteristics of respondents

As observed in Table 1, a total of 720 participants were included in the study. The majority were female (60.3%), aged 18–29 years (52.1%), and held at least a university degree (68.4%). Vitamin C consumption was reported by 73.6% of participants, with the primary source being natural foods such as fruits and vegetables (82.5%). Supplement use was more common among females and older age groups.

**Table 1:** Sociodemographic profile of study participants in the UAE (*n* = 720).

**Demographic factors**	**Frequency (*n*)**	**Percent (%)**
**Gender**		
Male	100	13.9
Female	620	86.1
**Nationality**		
UAE citizen	436	60.6
UAE resident	284	39.4
**Age**		
18–29 years old	544	75.6
30–39 years old	90	12.5
40–49 years old	41	5.7
50+ years old	45	6.3
**Education level**		
High school	255	35.4
Diploma	53	7.4
Undergraduate degree	313	43.5
Post graduate degree	79	11.0
Other	20	2.8
**Consumption of healthy diet**		
Yes	20	2.8
No	253	35.1
**Regular exercise**		
Yes	165	22.9
No	555	77.1
**Consuming of VC**		
Yes	286	39.7
No	434	60.3

Diet and 77.1% not engaging in regular exercise. When asked whether they consume VC and from which source, the majority (88.2) consume VC, Of those, 57.9% obtained VC from natural sources, such as fruits and vegetables, while 53.6% reported using VC supplements. Notably, 11.8% of participants did not consume VC.

### 3.2 Participants' knowledge and awareness of VC

As presented in Table 2, participants demonstrated varying levels of knowledge and behaviors related to VC. The results showed that only 32.5% correctly identified raw citrus fruits as high in VC, while 60.7% were unsure, reflecting limited awareness of VC-rich foods. Awareness of the side effects of VC deficiency was low, with only 11.1% aware, while 59.6% lacked knowledge and 29.4% were unsure. Similarly, knowledge of overdose risks was limited, with just 31.4% demonstrating awareness. However, 65.4% of participants were familiar with the antioxidant benefits of vitamin C, indicating some understanding of its health-promoting properties. Knowledge of the recommended daily allowance (RDA) for VC was particularly low, with only 19.3% aware. These gaps highlight the need for enhanced educational efforts to improve understanding of VC's role in health.

**Table 2:** Knowledge and awareness of vitamin C and its health effects among participants (*n* = 720).

**Knowledge No**.	**Knowledge questions**	**I know *N* (%)**	**I don't know *N* (%)**	**Not sure *N* (%)**
K1	Raw citrus fruits are very high in VC	234 (32.5)	49 (6.8)	437 (60.7)
K2	Side effects of VC deficienc	79 (11.1)	429 (59.6)	212 (29.4)
K3	Side effects of VC overdose	226 (31.4)	93 (13)	401 (55.6)
K4	Benefits of VC	152 (21.1)	68 (9.4)	500 (69.5)
K5	VC as an antioxidant	471 (65.4)	201 (27.9)	48 (6.7)
K6	RDA of VC^*^	139 (19.3)	471 (65.4)	110 (15.3)
Total		30.1%	29.8%	39.5%

^*^RDA: Recommended Daily Allowance of Vitamin C.

### 3.3. VC consumption before and after the COVID-19 pandemic

As shown in Table 3, prior to the COVID-19 pandemic, the majority of participants (60.3%) did not consume vitamin C (VC) regularly. Among those who did, intake was generally low, with the highest proportion consuming VC less than once per week (42.3%), followed by once per week (23.4%) and 2–3 times per week (15.0%).

**Table 3:** Vitamin C consumption practices before and during the COVID-19 pandemic (*n* = 720).

**Practices**	**Frequency (*n*)**	**Percent (%)**
**Consumption of VC before the COVID-19 pandemic**		
Less than 1 time per week	121	42.3
1 time per week	67	23.4
2–3 times per week	43	15.0
4–5 times per week	30	10.5
6–7 times per week	19	6.6
More than 6–7 times per week	6	2.1
**Variation of VC intake since the start of COVID-19 pandemic**		
Increase	115	40.2
Decrease	30	10.5
Stayed the same	141	49.3
**Change in consumption of VC during the COVID-19 pandemic**		
Less than 1 time per week	17	11.7
1 time per week	18	12.4
2–3 times per week	58	40.0
4–5 times per week	32	22.1
6–7 times per week	15	10.3
More than 6–7 times per week	5	3.4
**Started using VC during COVID-19 pandemic**		
Yes	232	53.5
No	202	46.5
**Reasons behind not consuming any VC supplements**		
No reason specified	18	10.7
Believe that there is no need or not necessary to consume Vit C	60	35.5
Uses other methods for acquiring Vit C	31	18.3
Not aware of certain aspects of Vit C	7	4.1
Does not like consuming Vit C supplements	11	6.5
Multifactorial reasons behind not consuming Vit C	7	4.1
Other	35	20.7
**Consumption of VC during the COVID-19 pandemic**		
Less than 1 time per week	67	29.0
1 time per week	42	18.2
2–3 times per week	61	26.4
4–5 times per week	31	13.4
6–7 times per week	24	10.4
More than 6–7 times per week	6	2.6

Among pre-pandemic VC users, nearly half (49.3%) reported no change in their intake during the pandemic, while 40.2% reported an increase. The most common post-pandemic intake frequency among these participants was 2–3 times per week (40.0%), followed by 4–5 times (22.1%), and once per week (12.4%).

For those who did not consume VC prior to the pandemic, the main reason reported was the belief that supplementation was unnecessary (35.5%), followed by other reasons (20.7%) and the use of alternative sources of VC (18.3%). After the onset of the pandemic, 53.5% of these participants began taking VC supplements, although their consumption remained relatively low, with the largest group reporting intake less than once per week (29.0%) or 2–3 times per week (26.4%).

These findings suggest a noticeable shift toward increased VC supplementation during the COVID-19 pandemic, particularly among new users; however, the overall intake frequency remained modest for many participants.

### 3.4. Changes in VC consumption before and during the COVID-19 pandemic

Table 4 presents the pandemic resulted in a statistically significant increase in VC intake among participants (*p* < 0.001). Despite this increase, no significant association was found between VC consumption and healthy lifestyle habits, such as regular exercise (*p* = 0.631). This indicates that the observed changes in VC consumption were likely driven by pandemic-related health concerns rather than broader lifestyle modifications. The increased focus on VC during the pandemic represents an opportunity for public health initiatives to encourage sustained improvements in dietary habits.

**Table 4:** Statistical comparison of vitamin C consumption before and during the COVID-19 pandemic (*n* = 720).

**Practice factors**	***N***	**MEAN ±SD**	**df**	***p*-Value**
Change in consumption of VC before and during the COVID-19 pandemic	145	0.093 ± 0.54	25	< 0.001^*^
Consumption of VC before the COVID-19 pandemic	286	0.317 ± 0.23	1	0.474
Consumption of VC during the COVID-19 pandemic	434	0.41 ± 0.49	1	0.631

^*^Significant at *p* < 0.05.

### 3.5. Participants' attitudes toward VC

As per Table 5, Participants expressed diverse attitudes toward CV consumption (A1). A majority of participants (81.9%) believed that overconsumption of VC is harmful, reflecting awareness of its potential risks (A2), most of respondents (85.8%) thought that VC aids recovery from COVID-19, but only 45.7% believed it could prevent the infection (A3). These mixed perceptions highlight the need for clearer communication about VC's role in immune support.

**Table 5:** Attitudes toward vitamin C during the COVID-19 pandemic (*n* = 720).

**Opinions on VC**	**Response**	**Frequency (*n*)**	**Percent (%)**
Do you feel that consuming too much VC might be harmful (A1)?	Yes	590	81.9
	No	130	18.1
Do you believe that the taking of VC when a person have COVID-19	Yes	618	85.8
positive help speed up their recovery (A2)?	No	102	14.2
Do you feel that VC might potentially protect	Yes	329	45.7
yourself of COVID-19 (A3)	No	391	54.3

### 3.6 Correlation between demographic factors, knowledge, and attitudes toward vitamin C

Table 6 presents the association between demographic variables and participants' knowledge and attitudes toward vitamin C (VC). A statistically significant difference in knowledge scores was found across age groups (*p* = 0.015), with participants aged 18–29 showing higher mean scores compared to older groups. No significant differences were observed in knowledge scores by gender (*p* = 0.494), nationality (*p* = 0.066), or education level (*p* = 0.513), indicating that age is the primary demographic factor associated with VC knowledge. Attitudes toward VC also varied across demographic groups. The majority of participants (81.9%) believed that excessive intake of VC may be harmful (A1), and most (85.8%) believed it supports recovery from COVID-19 (A2), though less than half (45.7%) believed it could prevent infection (A3). Attitude A1 (concern over harm) was significantly associated with gender (*p* < 0.001) and nationality (*p* = 0.012), while A2 (recovery benefit) showed a significant association with age (*p* = 0.031). Attitude A3 (protective effect) was significantly associated with age (*p* = 0.002) and gender (*p* = 0.026). Education level did not show a statistically significant relationship with any of the three attitude measures (A1–A3). These findings suggest that age, gender, and nationality influence both knowledge and attitudes toward VC, while education level appears to have limited impact.

**Table 6:** Association between demographics and knowledge and attitude scores (*n* = 720).

**Demographic variable**	**Group**	***N***	**Knowledge Mean ±SD**	**Attitude A1 Mean ±SD**	**Attitude A2 Mean ±SD**	**Attitude A3 Mean ±SD**	***p*-Value (K/A1/A2/A3)**
Age	18–29	544	9.57 ± 4.75	0.83 ± 0.38	0.84 ± 0.37	0.43 ± 0.49	0.015^*^/0.217/0.031^*^/0.002^*^
	30–39	90	7.73 ± 2.81	0.83 ± 0.38	0.89 ± 0.32	0.46 ± 0.50	
	40–49	41	8.80 ± 4.90	0.78 ± 0.42	0.95 ± 0.22	0.56 ± 0.50	
	50+	45	9.36 ± 4.86	0.71 ± 0.46	0.92 ± 0.21	0.71 ± 0.46	
Gender	Male	100	9.46 ± 5.20	0.68 ± 0.47	0.85 ± 0.36	0.56 ± 0.50	0.494/ < 0.001^*^/0.797/0.026^*^
	Female	620	9.41 ± 4.50	0.84 ± 0.37	0.86 ± 0.35	0.44 ± 0.50	
Nationality	UAE Citizen	436	9.05 ± 4.35	0.85 ± 0.36	0.85 ± 0.36	0.46 ± 0.50	0.066/0.012^*^/0.355/0.671
	UAE Resident	284	10.01 ± 4.93	0.77 ± 0.42	0.87 ± 0.33	0.45 ± 0.50	
Education Level	High School	255	9.64 ± 4.54	0.80 ± 0.40	0.88 ± 0.33	0.43 ± 0.50	0.513/0.276/0.750/0.433
	Diploma	53	10.13 ± 5.14	0.77 ± 0.42	0.38 ± 0.38	0.42 ± 0.50	
	Undergraduate	313	9.16 ± 4.47	0.85 ± 0.36	0.84 ± 0.36	0.47 ± 0.50	
	Postgraduate	79	9.22 ± 5.32	0.77 ± 0.42	0.87 ± 0.34	0.51 ± 0.50	
	Other^**^	20	9.65 ± 4.61	0.85 ± 0.37	0.85 ± 0.37	0.60 ± 0.50	

^**^Other included having an experienced certificate, being a graduate but did not specify the degree and did not specify their education level.

^*^Significant at p < 0.05.

## 4 Discussion

This study is one of the first conducted in the UAE to assess knowledge, attitudes, and prevalence of VC intake among the general population during the COVID-19 pandemic, while also comparing behaviors before and during the health crisis. Vitamin C plays a role in supporting immune function during COVID-19 infection, particularly in aiding recovery from active illness (1). However, many individuals may lack awareness of vitamin C–rich foods or the recommended supplementation levels needed to achieve such benefits (18). This is reflected in our findings, which show low knowledge scores in both categories.

The findings reveal a noteworthy trend: a significant increase in VC consumption during the pandemic, particularly among individuals who were not regular users prior to the outbreak. This rise appears to be driven by the widely held perception of VC as a preventive and therapeutic agent against respiratory infections (14, 16, 19).

In terms of the study population, the majority of respondents were female, UAE nationals, and individuals aged 18–29 years. The predominance of females and UAE nationals in this study, which differs from the expected demographic distribution within the UAE, can be attributed to the snowball a non-probability, self-selected convenience sampling method used. This sampling approach often leads to the recruitment of individuals with similar demographic backgrounds. The predominance of individuals aged 18–29 years is consistent with the general age distribution in the UAE population. This can also be attributed to the study's use of electronic devices, which are more readily accessible and commonly utilized by younger individuals (20). The higher proportion of female participants in our study aligns with findings from other studies conducted in the UAE during the same period, where the majority of participants were also female (19, 21–24).

In terms of participants' lifestyles, the majority reported poor dietary habits and limited physical activity, which is consistent with the general demographic trends in the UAE. According to the UAE National Health Survey, approximately 27.8% of the UAE national population is obese, accounting for both genders (20). The data is consistent with other research conducted during the COVID-19 pandemic, which highlighted lifestyle habits in the UAE. A 2023 study by AlBlooshi showed an increase in food consumption and a decrease in physical activity among the UAE population during the COVID-19 period (25). Furthermore, these findings align with multiple international studies, including those from Spain, Brazil, and Italy, which reported a rise in weight among the global population during the pandemic. This increase is believed to be linked to poor diet and a lack of regular exercise or normal daily activities, contributing to the high prevalence of unhealthy habits within our population (26, 27). The study found that despite increased consumption, only 30.1% of participants exhibited adequate knowledge about VC's functions, sources, and safe use. This suggests that rising usage was not necessarily paired with an understanding of recommended daily allowances or potential side effects of overuse. Indeed, a significant proportion (81.9%) acknowledged that excessive VC intake could be harmful, yet misconceptions persisted regarding its protective role against infection (45.7% believed it could prevent COVID-19).

Our study found that younger participants had significantly higher knowledge scores than other age groups. This could be attributed to the fact that younger individuals are more likely to encounter information through online sources and digital media, which has been shown to enhance health literacy and access to nutritional information among younger populations (28, 29). Similar findings were reported in studies by Saeed et al. (19) and Alowais and Selim (13), where younger participants also demonstrated higher knowledge compared to older groups. Our study found no significant correlation between age groups and VC consumption rates, which differs from a similar study that reported a significant association for individuals aged ≥50 years (*p* < 0.001). This discrepancy may be attributed to differences in study populations; their sample included a larger proportion of individuals aged ≥50 years (20%) compared to ours (6.9%) (30).

The demographic factors, including age, sex, and nationality, significantly influenced attitudes toward VC. Age was strongly associated with beliefs about VC's role in enhancing recovery (*p* = 0.031) and providing protection against COVID-19 (*p* = 0.002).

In our study, the increase in VC consumption observed in both groups within our study, particularly the shift from low or no intake to higher consumption rates, likely reflects a response to the COVID-19 pandemic. The data demonstrate that 53.5% of participants who were previously non-consumers began taking VC during the pandemic. Moreover, among prior users, a substantial portion reported increasing their intake frequency. These shifts suggest that public concern about immunity, amplified by health campaigns, social media discourse, and word-of-mouth recommendations, likely influenced consumer behavior. This aligns with findings from Lebanon and Saudi Arabia, where VC usage similarly surged in response to heightened health awareness during the pandemic (14, 30).

This pattern also reflects a reactive, rather than preventive, approach to supplementation, wherein individuals responded to a global threat by turning to accessible, over-the-counter solutions. While vitamin C (VC) has well-established roles in supporting immune function (1, 31), its actual efficacy in preventing COVID-19 remains limited and context-dependent (32–34). This discrepancy between public perception and scientific evidence underscores the need to guide communities with accurate, evidence-based information.

A majority of participants expressed concern about potential harm from overconsumption (81.9%), agreed that vitamin C aids in recovery from COVID-19 (85.8%), and a smaller portion believed it provides protection against infection (45.7%). These findings reflect a mix of evidence-based understanding and misconceptions that have been reported in similar studies conducted in the UAE and internationally (13, 14). Moreover, our analysis demonstrated that attitudes varied significantly by demographic factors: younger participants and females were more likely to hold positive views about vitamin C's protective and therapeutic effects, consistent with prior studies showing that women and younger individuals tend to report more proactive health behaviors and supplement use (13, 24). Nationality also influenced beliefs about safety concerns, possibly reflecting cultural perceptions and traditional health practices common in Middle Eastern and South Asian communities (14). Nationality influenced beliefs about the side effects of VC overconsumption (*p* = 0.012). This finding may reflect the cultural background of the UAE's population, which is predominantly composed of individuals from Middle Eastern and Asian regions, where natural remedies, including VC, are widely valued (14). Contrary to findings from an Egyptian study, which reported a positive correlation between fear scores and VC consumption, our study found no such association. This divergence underscores the need for future research to explore how fear and other psychological factors influence VC intake (30).

An important observation in our study was the low level of awareness regarding VC among the participants. This could be attributed to the widespread availability of VC-rich foods in the UAE, which may reduce the perceived need for detailed knowledge about its dietary sources or the risks of deficiency. Additionally, VC is often fortified in common food products, further diminishing public awareness. However, this knowledge gap is concerning, particularly for at-risk populations such as individuals with eating disorders, G6PD deficiency, or a susceptibility to kidney stones. Educational initiatives are essential to address these gaps and promote informed decision-making regarding VC consumption (14).

These findings highlight the need for targeted educational interventions. Healthcare professionals, especially pharmacists who are often the first point of contact for individuals purchasing supplements should be equipped to counsel the public. Key messages should emphasize the actual benefits and limitations of VC in infection prevention and recovery (1, 31), Recommended daily allowances and safe upper limits (35), the Risks associated with mega dosing, especially for vulnerable groups (e.g., individuals with kidney conditions or G6PD deficiency) (35, 36), highlighting the importance of obtaining nutrients from balanced diets rather than supplements alone (37).

The observed increase in VC use during the pandemic presents both a challenge and an opportunity. It reflects the public's willingness to engage in health-promoting behaviors but also reveals gaps in understanding. Strengthening the communication of evidence-based nutrition guidance through pharmacists, health campaigns, and digital media can help transform reactive behaviors into informed, preventive health practices.

## 5 Limitations

This study has several limitations. First, the data were collected through a self-reported, online questionnaire, which may have introduced inaccuracies due to participants' varying interpretations of the questions and potential recall bias. Although efforts were made to ensure clarity, the absence of interviewer guidance may have led to misinterpretation of some items. Second, because the survey was administered online, only individuals with internet access and basic digital literacy could participate, potentially excluding certain demographic groups and limiting the generalizability of the findings. Third, the study relied on a non-probability, self-selected sample, which may have led to sampling bias and overrepresentation of specific demographic groups such as young adults and females.

In addition, respondents may have been inclined to provide socially desirable answers, particularly regarding health behaviors, despite the assurances of anonymity. While informed consent was obtained and confidentiality emphasized, the lack of validated scoring procedures and standardized psychometric tools could have further contributed to response bias.

To mitigate these issues in future research, we recommend the use of validated and reliability-tested instruments, probability-based sampling methods to enhance representativeness, and improved strategies to minimize social desirability bias such as anonymized offline data collection or randomized response techniques. These adjustments will help produce more accurate and generalizable insights.

## 6 Conclusion

In conclusion, this study assessed the knowledge, attitudes, and prevalence of vitamin C intake among the general population in the UAE during the COVID-19 pandemic. While many participants reported consuming vitamin C, particularly through natural sources and supplements, knowledge regarding its health benefits, recommended daily allowance, and potential risks remained limited in key areas. Attitudinal responses were mixed: most participants believed vitamin C aids in COVID-19 recovery and acknowledged potential risks of overconsumption, yet fewer believed it could prevent infection.

Significant differences in knowledge and attitudes were observed across demographic groups, with age, gender, and nationality influencing responses. These findings highlight the need for targeted educational initiatives to promote accurate, evidence-based information on vitamin C use. Public health campaigns and media-based outreach may play a crucial role in improving awareness and encouraging informed decision-making regarding supplementation and dietary intake.

## Data Availability

The original contributions presented in the study are included in the article/supplementary material, further inquiries can be directed to the corresponding author.
